# 胸腺瘤合并重症肌无力患者预后的临床研究

**DOI:** 10.3779/j.issn.1009-3419.2018.01.01

**Published:** 2018-01-20

**Authors:** 东风 袁, 志涛 谷, 光辉 梁, 文涛 方, 印 李, 中国胸腺肿瘤研究协作组

**Affiliations:** 1 450008 郑州，郑州大学附属肿瘤医院胸外科 Department of Thoracic Surgery, Affiliated Cancer Hospital of Zhengzhou University, Zhengzhou 450008, China; 2 200030 上海，上海交通大学附属上海胸科医院 Shanghai Chest Hospital, Affiliated to Shanghai Jiaotong University, Shanghai 200030, China

**Keywords:** 胸腺瘤, 重症肌无力, 胸腺扩大切除, 预后, Thymoma, Myasthenia gravis, Extended thymectomy, Prognosis

## Abstract

**背景与目的:**

胸腺瘤常伴发重症肌无力（myasthenia gravis, MG），但是这些患者行胸腺切除的预后与MG的关系尚不明确。本研究旨在探讨影响胸腺瘤合并MG患者预后的因素。

**方法:**

回顾性分析中国胸腺瘤协作组（Chinese Alliance for Research of Thymoma, ChART）数据库1992年-2012年875例随访20年资料完整的胸腺瘤病例，分析世界卫生组织（World Health Organization, WHO）组织学分型、Masaoka分期、术后辅助治疗与MG及预后的关系。

**结果:**

胸腺瘤WHO组织学分型与MG有相关性，差异有统计学意义（χ^2^=24.908, *P* < 0.001）。MG发生率为22.7%，其中B2型（58/178, 32.58%） > B3型（65/239, 27.20%） > B1型（27/132, 20.45%） > AB型（43/267, 16.10%） > A型（6/59, 10.17%），Masaoka分期与MG无相关性（χ^2^=1.365, *P*=0.714）。生存分析表明WHO分型、Masaoka分期与预后有关（*P* < 0.05），而是否合并MG（χ^2^=0.113, *P*=0.736）、是否行胸腺扩大切除（χ^2^=1.548, *P*=0.213）、术后辅助放疗（χ^2^=0.380, *P*=0.538）与预后无相关，术后辅助化疗与差的预后相关（χ^2^=14.417, *P* < 0.001）。是否行胸腺扩大切除与MG的疗效有相关性（χ^2^=24.695, *P* < 0.001）。

**结论:**

胸腺瘤患者是否合并MG和是否行胸腺扩大切除与预后无相关性，胸腺扩大切除可改善MG患者的疗效。

## Abstract (en)

**Background and objective:**

Thymoma is frequently associated with myasthenia gravis (MG). However, whether MG is a factor for the outcome of patients with thymoma following complete thymectomy remains unknown. The aim of this study is to investigate the effect of thymoma with MG prognostic factors.

**Methods:**

A retrospective analysis of The Chinese Alliance for Research in Thymomas (ChART) database within 1992-2012 complete cases 875 cases, 20 years follow-up data analysis thymic tumor tissue type credits and MG, Masaoka staging and prognosis, postoperative adjuvant therapy and relationship with the prognosis of surgical removal of the way.

**Results:**

Thymic tumor tissue type credit has correlation with MG, difference was statistically significant (χ^2^=24.908, *P* < 0.001). MG: incidence of B2 type (58/178, 32.58%) > B3 type (65/239, 27.20%) > B1 (27/132, 20.45%) > AB (43/267, 16.10%) > type A, 10.17% (6/59), Masaoka stage has no correlation with MG (χ^2^=0.365, *P*=1.365). Survival analysis showed that the WHO classification, Masaoka stage associated with prognosis (*P* < 0.05), and whether the merger MG (χ^2^=0.113, *P*=0.736), postoperative adjuvant radiotherapy (χ^2^=0.380, *P*=0.538) has nothing to do with the prognosis, postoperative adjuvant chemotherapy is associated with poor prognosis (χ^2^=14.417, *P* < 0.001). Whether has nothing to do with the prognosis of the thymus resection (χ^2^=1.548, *P*=1.548), whether the whole correlated with the curative effect of thymus excision with MG (χ^2^=24.695, *P* < 0.001).

**Conclusion:**

Thymoma patients with MG and extended thymectomy have no correlation with prognosis. Extended thymectomy can improve the effect of MG patients.

所有胸腺瘤都是恶性肿瘤^[1]^，其发病率未知。占前纵隔肿瘤的47%^[2-3]^。胸腺瘤可发生于任何年龄，集中发病年龄为35岁-70岁。男女发病率大致相等，老年人中女性发病率高于男性^[4]^。其增殖缓慢，常与良性肿瘤混淆。胸腺瘤约1/3合并重症肌无力（myasthenia gravis, MG），外科切除可改善大多数MG患者的临床症状。德国学者最早提出了胸腺瘤与MG之间有相关关系这一论点，但到目前为止其原因还不清楚^[5]^。本文通过回顾分析ChART数据库1992年-2012年20年胸腺瘤患者资料，有完整资料者875例，按2004年世界卫生组织（World Health Organization, WHO）组织学分型标准进行分型，探讨胸腺瘤合并MG患者的组织学分型、临床分期特点、临床切除状况、术后辅助治疗情况及其预后。

## 1 资料与方法

### 1.1 临床资料

本组行胸腺瘤切除术875例。男性439例，女性436例；年龄15岁-83岁，平均年龄（50.9±12.0）岁。其中胸腺瘤合并MG 199例，男性99例，女性100例；胸腺瘤未合并MG 676例，男性340例，女性336例。

### 1.2 方法

#### 1.2.1 组织学分型

按WHO（2004）分型标准进行分型，其中A型59例，AB型267例，B1型132例，B2型178例，B3型239例。

#### 1.2.2 Masaoka分期

Ⅰ期：肉眼见完整的包膜，无镜下包膜外侵犯；Ⅱ期：镜下侵出包膜或肉眼见侵犯纵隔脂肪组织或纵隔胸膜；Ⅲ期：肉眼见侵犯临近结构（如：心包、大血管或肺）；Ⅳa期：胸膜腔播散（胸膜或心包转移)；Ⅳb期：淋巴或血行转移，胸腔外播散（以骨转移最为常见）。本文病例其中Ⅰ期400例，Ⅱ期209例，Ⅲ期210例，Ⅳ期56例。

#### 1.2.3 手术切除方式

包括胸腺扩大切除和胸腺瘤切除。胸腺扩大切除包括全部胸腺组织和前纵隔脂肪组织（上至甲状腺下极，下达膈肌，两侧边界为膈神经），胸腺瘤切除是仅仅完整切除肿瘤，未切除全部胸腺组织和前纵隔脂肪组织。

### 1.3 统计学分析

针对胸腺瘤各组织学分型所伴随的MG、不同手术方式、Masaoka病理分期及术后辅助治疗等因素进行统计学分析。采用SPSS 19.0软件，进行卡方检验，生存分析采用*Kaplan-Meier*法，生存差异采用*Log-rank*法。*P* < 0.05差异有统计学意义。

## 2 结果

### 2.1 胸腺瘤组织学分型和临床特征

本组患者共875例，男性439例，女性436例，男：女=1.006：1，年龄15岁-83岁，平均年龄（50.9±12.0）岁。其中胸腺瘤合并MG为22.7%（199/875），胸腺瘤未合并MG为87.3%（676/875）；胸腺瘤组织学分型与MG发生率之间差异有统计学意义（*P* < 0.001），见表 1。

**1:** 胸腺瘤病理组织学分类与临床分期的关系 Relationship between histopathological classification and clinical stages

WHO classification	Masaoka staging			
	Ⅰ	Ⅱ	Ⅲ	Ⅳ
A (*n*=59)	38	14	7	0
AB (*n*=267)	172	69	24	2
B1 (*n*=132)	62	37	27	6
B2 (*n*=178)	69	42	50	17
B3 (*n*=239)	59	47	102	31
Total (*n*=875)	400	209	210	56
χ^2^=155.209, *P* < 0.001.				

### 2.2 胸腺瘤组织学分型与Masaoka病理分期的关系

胸腺瘤组织分型与Masaoka病理分期具有相关性。差异有统计学意义（*P* < 0.001），见表 1。

### 2.3 MG与WHO组织学分型的关系

按WHO组织学分型本组合并有MG患者为：A型6例（10.17%），AB型43例（16.10%），B1型27例（20.45%），B2型58例（32.58%），B3型65例（27.20%）。胸腺瘤组织学分型与MG有相关性，差异有统计学意义（χ^2^=24.908, *P* < 0.001），见表 2。

**2:** WHO病理分型与MG关系 Relationship between WHO classification and MG

WHO classification	MG	Non-MG
A (*n*=59)	6	53
AB (*n*=267)	43	224
B1 (*n*=132)	27	105
B2 (*n*n=178)	58	120
B3 (*n*=239)	65	174
Total (*n*=875)	199	676
χ^2^=24.908, *P* < 0.001.		

### 2.4 MG与Masaoka病理分期的关系

根据Masaoka病理分期本组中合并有MG的患者为：Ⅰ期85例（21.25%）、Ⅱ期49例（23.44%）、Ⅲ期53例（25.24%）、Ⅳ期12例（21.43%）。胸腺瘤是否伴有MG与Masaoka病理分期无相关性（χ^2^=1.365, *P*=0.714），见表 3。

**3:** Masaoka分期与MG关系 Relationship between Masaoka staging and MG

Masaoka staging	MG	Non-MG
Ⅰ (*n*=400)	85	315
Ⅱ (*n*=209)	49	160
Ⅲ (*n*=210)	53	157
Ⅳ (*n*=56)	12	44
Total (*n*=875)	199	676
χ^2^=1.365, *P*=0.714.		

### 2.5 MG与是否行胸腺扩大切除的关系

本组合并MG共199例，所有资料完整192例，其中胸腺扩大切除者170例，有效（MG好转）164例，无效（MG无变化或恶化）6例，有效率96.47%；胸腺瘤切除者22例，有效（MG好转）15例，无效（MG无变化或恶化）7例，有效率68.18%。总的有效率为93.23%。行胸腺扩大切除患者MG治疗效果优于胸腺瘤切除患者，两者MG治疗的有效率差异有统计学意义（χ^2^=24.695, *P* < 0.001）。

### 2.6 MG与术后辅助治疗的关系

199例MG患者中行术后辅助化疗13例，行术后辅助放疗96例。进一步的分析显示术后辅助化疗、放疗并不能改善MG的疗效（χ^2^=1.618, *P*=0.218; χ^2^=1.529, *P*=0.376）。

### 2.7 生存分析

875例胸腺瘤患者总的5年生存率是0.89。与预后有关的因素的*Kaplan-Meier*生存分析结果见图 1-图 4。生存分析结果表明预后与临床分期（*P* < 0.05）、病理组织学分型（*P* < 0.05）、临床切除状态（*P* < 0.001）均相关，而是否合并MG（*P*=0.736）、是否行胸腺扩大切除（*P*=0.213）、术后辅助放疗（*P*=0.538）与预后无相关，术后辅助化疗与差的预后相关（*P* < 0.001）。而对于MG患者术后化疗或放疗并不影响其预后（*P*=0.150, *P*=0.424）。

**1:**
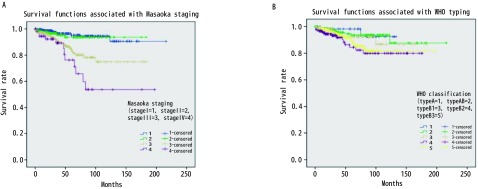
Massoka分期和WHO分型相关的生存曲线。A：不同Massoka分期生存差异有统计学意义（*P* < 0.05）；B：不同WHO分型生存差异有统计学意义（*P* < 0.05）。 Survival curves associated with Massoka staging and WHO typing. A: There was significant difference in survival rates among different Massoka stages (*P* < 0.05); B: There was significant difference in survival rates among different WHO subtypes (*P* < 0.05).

**2:**
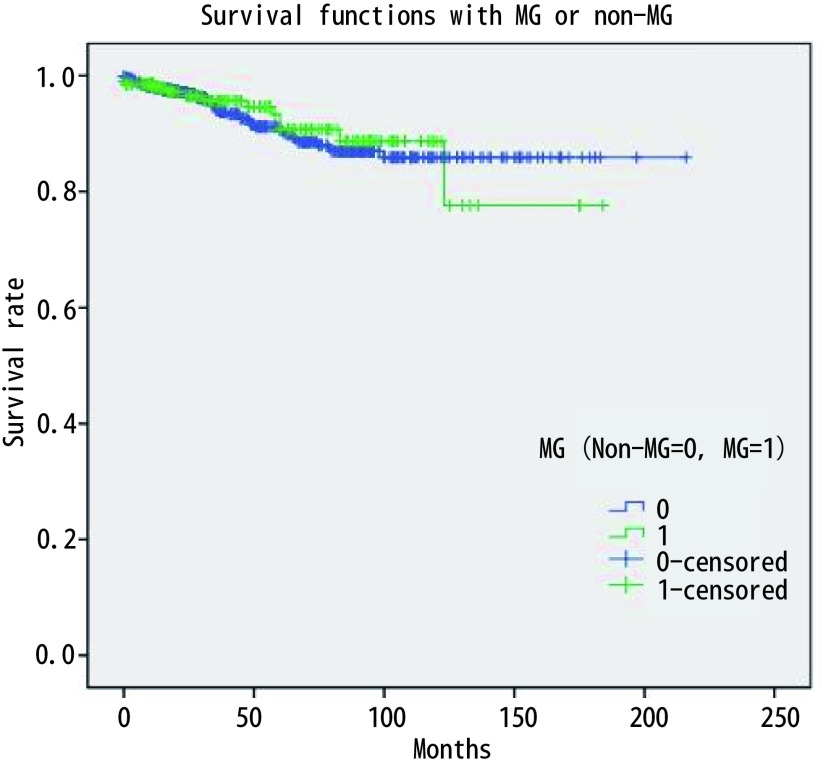
是否合并MG总体生存无明显差异（*P*=0.213） There was no significant difference in overall survival between MG and Non-MG (*P*=0.213)

**3:**
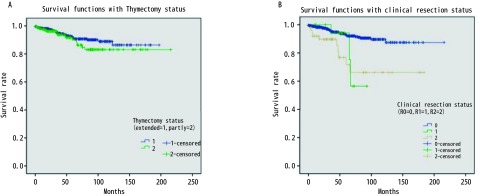
是否行胸腺扩大切除和临床切除状况相关的生存曲线。A：行胸腺扩大切除并不能改善胸腺瘤患者的预后（*P*=0.736）；B：不同临床切除状况的生存分析显示，R0切除患者预后相对较好（*P* < 0.001）。 Survival curves associated with extended thymectomy and clinical resection. A: Extended thymectomy does not improve the prognosis of patients with thymoma (*P*=0.736); B: Different clinical conditions resection and survival analysis showed that patients with R0 resection and relatively good prognosis (*P* < 0.001).

**4:**
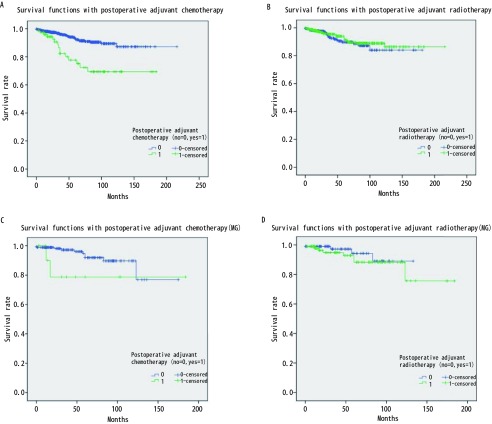
辅助化、放疗相关的生存曲线。A：行术后辅助化疗与未行术后辅助化疗患者的生存比较显示，未行术后辅助化疗患者预后相对较好（*P* < 0.001）；B:行术后辅助放疗与未行术后辅助放疗患者的生存比较显示，二者生存无差异（*P*=0.538）；C：MG患者术后行辅助化疗与未行术后辅助化疗的生存比较显示，二者生存无差异（*P*=0.150）；D: MG患者术后行辅助放疗与未行术后辅助放疗的生存比较显示，二者生存无差异（*P*=0.424）。 Survival curve associated with adjuvant chemotherapy and adjuvant radiotherapy. A: The survival of patients who received adjuvant chemotherapy and without adjuvant chemotherapy showed that the prognosis of patients who did not undergo adjuvant chemotherapy was relatively good (*P* < 0.001); B: Survival comparison between adjuvant radiotherapy and postoperative adjuvant radiotherapy in patients showed that there was no difference in survival between the two groups (*P*=0.538); C: Survival comparison between adjuvant chemotherapy and non adjuvant chemotherapy after MG showed that there was no difference in survival between the two groups (*P*=0.150); D: Survival comparison between adjuvant radiotherapy and postoperative adjuvant radiotherapy in MG patients showed that there was no difference in survival between the two groups (*P*=0.424).

## 3 讨论

胸腺瘤的预后较好，有研究报道其10年的总生存率达0.73（95%CI: 0.69-0.75）^[6]^。本研究875例胸腺瘤患者的5年总生存率为0.89。*Kaplan-Meier*生存分析结果显示Massoka分期、WHO组织学分型和临床切除状态为影响其预后的因素，而是否合并MG和术后辅助放疗并不影响胸腺瘤患者的预后，术后辅助化疗为不良预后因素。对MG治疗效果分析显示，胸腺扩大切除可以改善MG患者MG的疗效。之前有文献分析了ChART数据库中胸腺瘤合并MG患者的预后^[7]^，但其包括了胸腺癌的患者，因胸腺瘤和胸腺癌的生物学形态和临床预后有较大差别，一起分析会造成结果存在较大的偏倚。目前更多的学者主张在对胸腺肿瘤的治疗和预后进行分析时，应该把胸腺瘤和胸腺癌分开。胸腺瘤合并MG的患者的预后应该包括肿瘤学的生存结果和MG的治疗效果评价。本文排除了胸腺癌的患者，并且对MG的治疗效果进行了分析，结果更有说服力。

关于MG对胸腺瘤预后的影响尚无定论^[8, 9]^。以前的研究结果和最近的并不一致^[10-12]^。过去认为MG是胸腺瘤预后的不良因素，因为围术期肌无力危象等导致围术期死亡率增加。近些年来有学者认为MG症状有利于胸腺瘤的早诊早治，从而使手术完整切除率较高，患者预后反而较好^[12-14]^。本研究结果显示MG并不影响胸腺瘤患者的预后，MG与Massoka分期无相关性，MG与WHO组织学分型相关。本研究中MG的发生率B2型 > B3型 > B1型 > AB型 > A型，这与以前的研究结果一致^[15-17]^。有关术后辅助放疗至今仍有争议，有文献报道完整切除的胸腺瘤患者放疗并不能带来生存获益^[18]^。但也有专家认为对于完整切除的Massoka分期IIb期以上的胸腺瘤患者，术后辅助放疗虽然不能改善总生存，但可以改善无进展生存^[19]^。

胸腺瘤的切除与MG的疗效是一个有趣的现象。本研究结果显示胸腺瘤切除术后MG有效率为93.2%，行胸腺扩大切除术后MG有效率为68.2%。术后辅助放化疗被证明不能改变MG的疗效。以前的研究报道了术前未合并MG的患者术后可能发生MG，发生率有1.0%-28%^[20-25]^。这意味着胸腺瘤并不是MG发生的唯一原因，还有其他的机制参与MG的发生发展。胸腺的免疫调节作用依靠CD4^+^ T淋巴细胞^[26, 27]^。有学者认为胸腺瘤切除术后使这种免疫调节消失，导致B淋巴细胞活性增强和产生抗体增加^[28]^。另外有研究表明胸腺瘤周围脂肪组织中存在异位胸腺或微小胸腺瘤可能是导致术后发生肌无力或肌无力危象的原因^[24]^。因此胸腺扩大切除应为胸腺瘤合并MG患者的标准治疗，这不仅是由于肿瘤学的考虑而是可以改善MG的治疗效果。

本研究是回顾性分析，患者资料来自ChART数据库20年的资料。患者资料时间跨度较大，一些患者因为信息不全而被排除在外，这使我们的资料存在选择偏倚。

总之，胸腺瘤患者的预后与Massoka分期和WHO分型相关，而合并MG与术后辅助放疗对胸腺瘤的预后影响不大，术后辅助化疗是预后不良的因素。胸腺扩大切除并不影响胸腺瘤的预后，而会改善合并MG胸腺瘤患者的MG治疗效果。
